# Pseudoaneurysm of the mitral-aortic intervalvular fibrosa: a rare case after percutaneous transluminal coronary angioplasty

**DOI:** 10.1186/s12872-023-03512-4

**Published:** 2023-09-26

**Authors:** Debo Xie, Lulu Jiang, Jun Zhang, Xin Li, Yanli Guo

**Affiliations:** Department of Ultrasound, Southwest Hospital, Army Medical University (Third Military Medical University), Chongqing, 400038 China

**Keywords:** Pseudoaneurysm of the mitral-aortic intervalvular fibrosa, Percutaneous transluminal coronary angioplasty, Echocardiography

## Abstract

**Background:**

Pseudoaneurysm of the mitral-aortic intervalvular fibrosa (P-MAIVF) is an uncommon but potentially life-threatening condition. The most common pathogenic factors of P-MAIVF are infective endocarditis and surgical valve operation. Here, we report a rare case of P-MAIVF which occurred one year after percutaneous transluminal coronary angioplasty (PTCA).

**Case presentation:**

A 31-year-old man developed a P-MAIVF one year after PTCA. Transthoracic echocardiography (TTE) revealed a pseudoaneurysm between the aortic root and the left atrium. Three-dimensional transesophageal echocardiography (3D-TEE) clearly demonstrated the orifice of the pseudoaneurysm. This case was initially diagnosed by ultrasound, and the prognosis was good after surgical repair.

**Conclusions:**

We report a rare case of P-MAIVF that occurred one year after PTCA.

**Supplementary Information:**

The online version contains supplementary material available at 10.1186/s12872-023-03512-4.

## Background

**Fig. 1:**
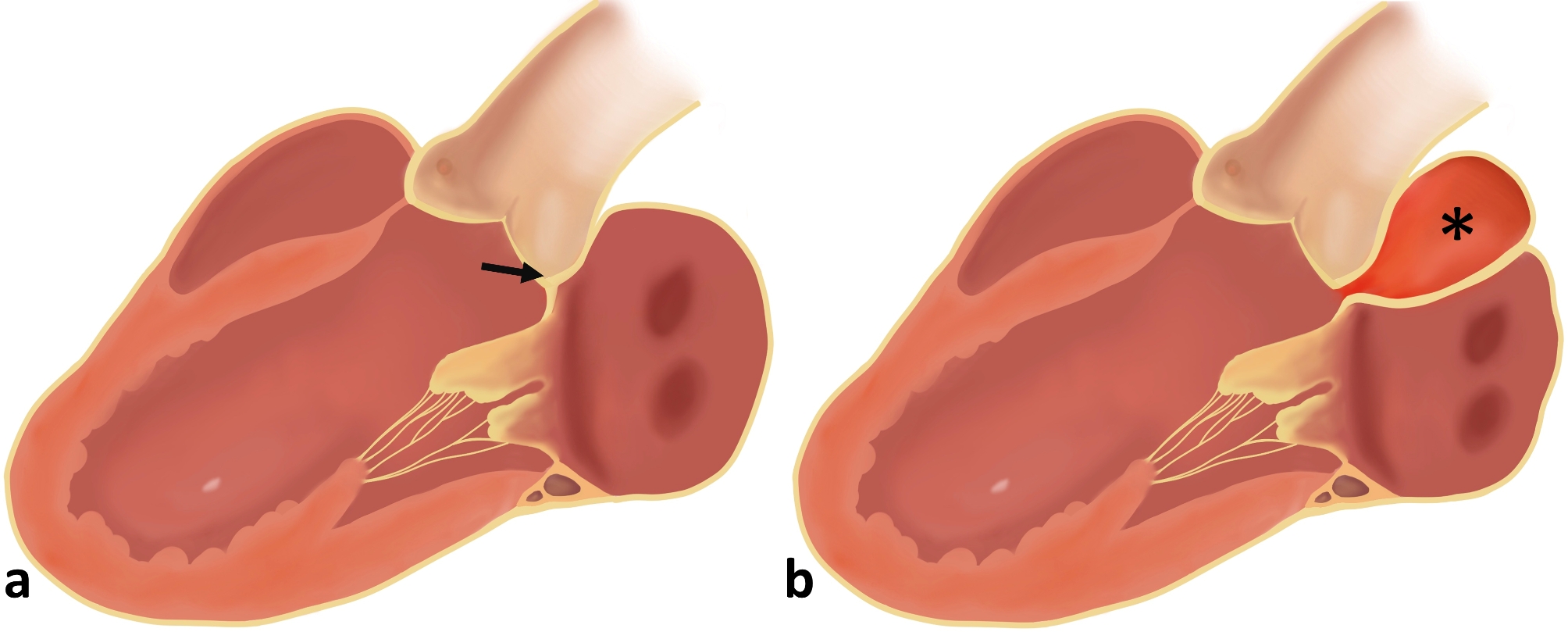
**a** The mitral-aortic intervalvular fibrosa (MAIVF) (arrow); **b** The pseudoaneurysm of the MAIVF (P-MAIVF) (*)

Mitral-aortic intervalvular fibrosa (MAIVF) is the region of fibrous tissue between the non-coronary cusp and the left coronary cusp of the aortic valve and anterior mitral leaflet. It is relatively avascular and more susceptible to injury and infection, which can cause abscesses or pseudoaneurysms (Fig. 1). In previous reports, pseudoaneurysm of the MAIVF (P-MAIVF) typically occurs after infective endocarditis or heart valve surgery [1]. In this report, we describe a rare case of P-MAIVF, which occurred one year after percutaneous transluminal coronary angioplasty (PTCA).

## Case presentation

A 31-year-old man was admitted to the emergency room a year ago with sudden chest pain for 3 h. He had a problematic smoking past. Acute myocardial infarction of inferior and posterior wall was revealed by the electrocardiogram. Blood tests showed cardiovascular troponin increased to 0.12 ng/ml and myoglobin reached 259 ng/ml. Transthoracic echocardiography (TTE) indicated mild regurgitation of the mitral valve and aortic valve and no abnormality was discovered in MAIVF. The patient was diagnosed with acute myocardial infarction. Emergency coronary angiography was performed 2 h after being hospitalized and revealed that the posterior descending branch was blocked. The patient received PTCA as an emergency, and the symptoms were relieved after the procedure (Video. 1–2). Before discharge, TTE revealed no abnormalities in MAIVF.

**Fig. 2:**
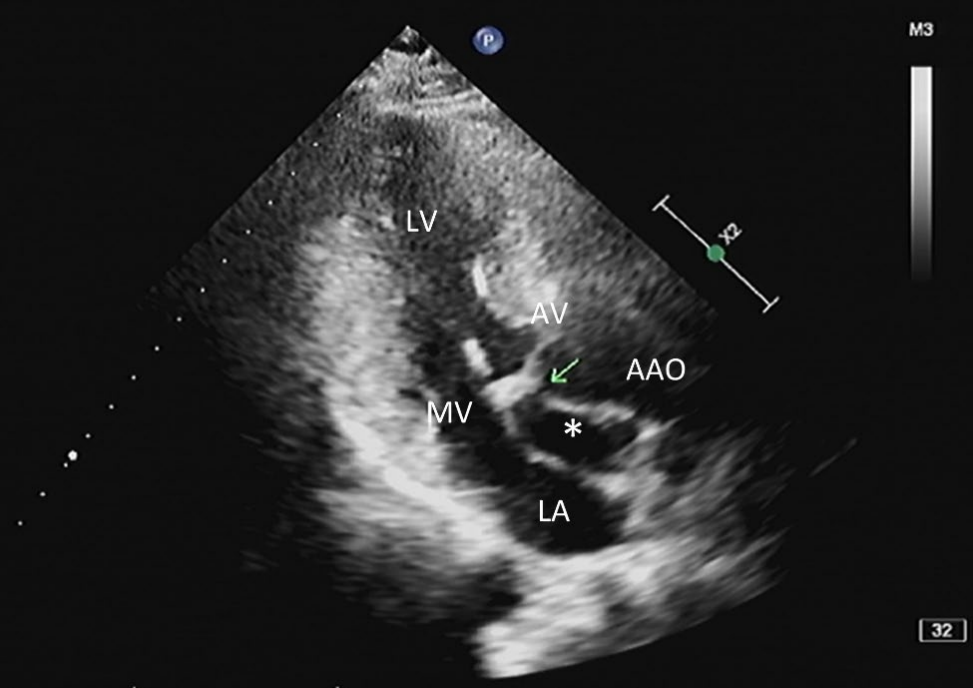
TTE image of P-MAIVF (*). LA = left atrium; LV = left ventricle; AAO = ascending aorta; AV = aortic valve; MV = mitral valve

**Fig. 3:**
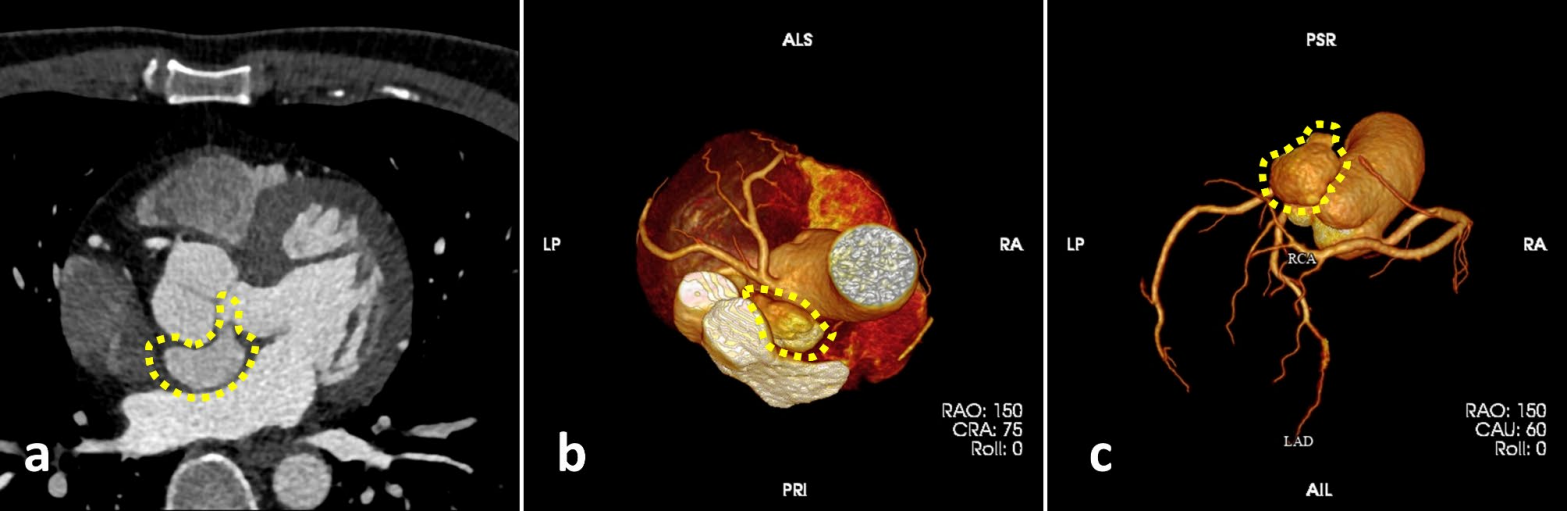
Contrast-enhanced CT image revealing the morphology of pseudoaneurysm. The yellow dotted line indicates the P-MAIVF.

**Fig. 4:**
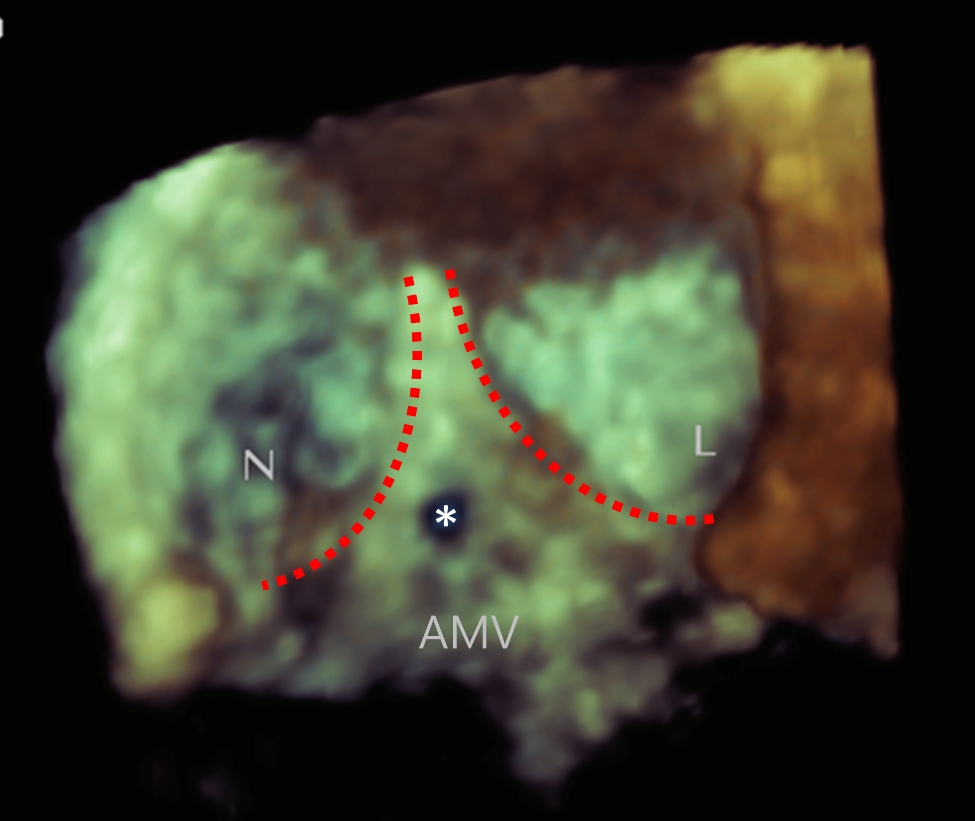
3D-TEE illustrating the orifice of P-MAIVF (*). N: Noncoronary aortic cusp; L: left coronary aortic cusp; AMV: anterior mitral valve; the red dotted line marks the aortic valve

**Fig. 5:**
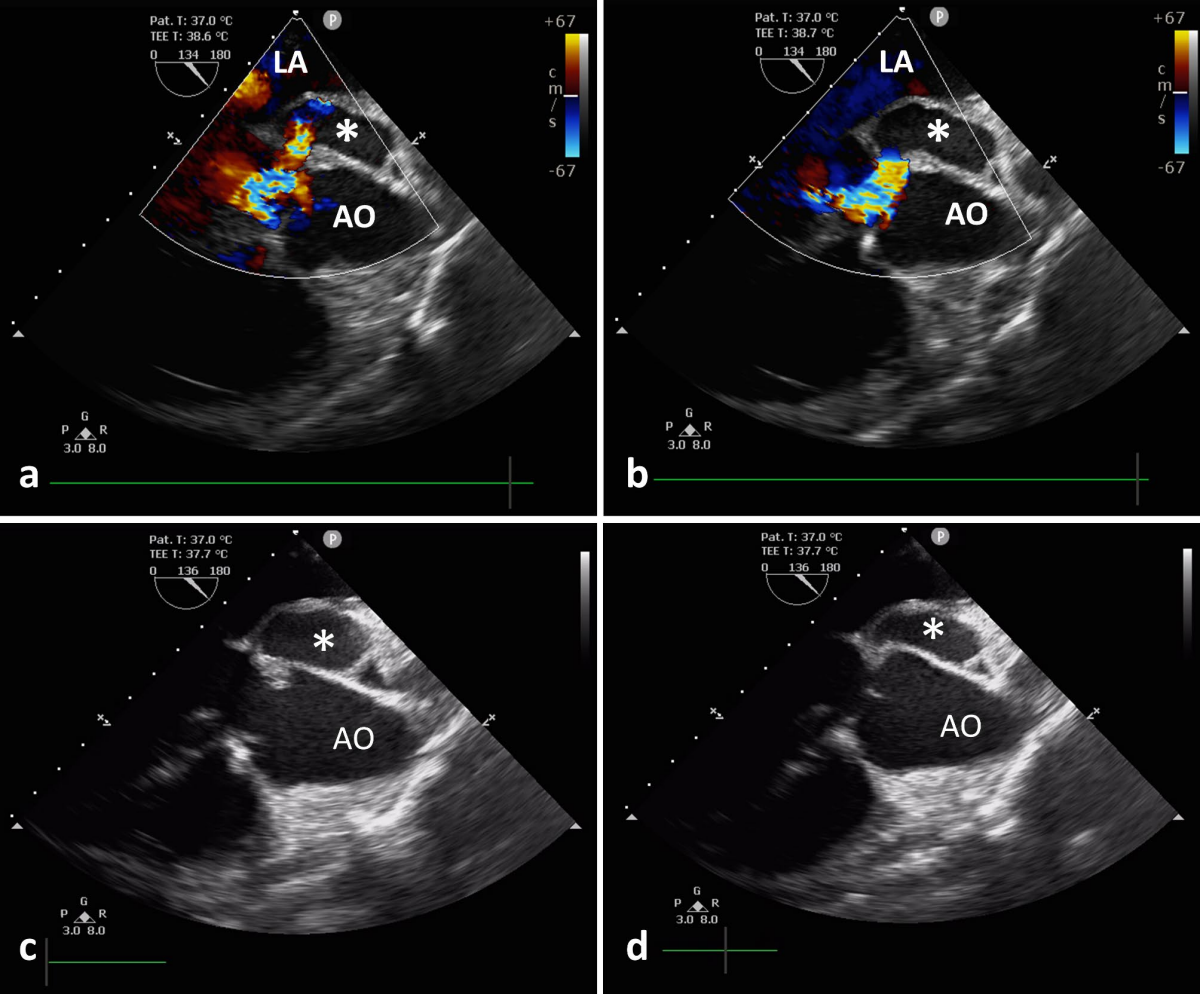
**a** CDFI shows the blood flow into the P-MAIVF (*) during systole; **b** CDFI shows the blood flow back into LVOT during diastole ; **c** P-MAIVF expanding during systole; **d** P-MAIVF contracting during diastole

**Fig. 6:**
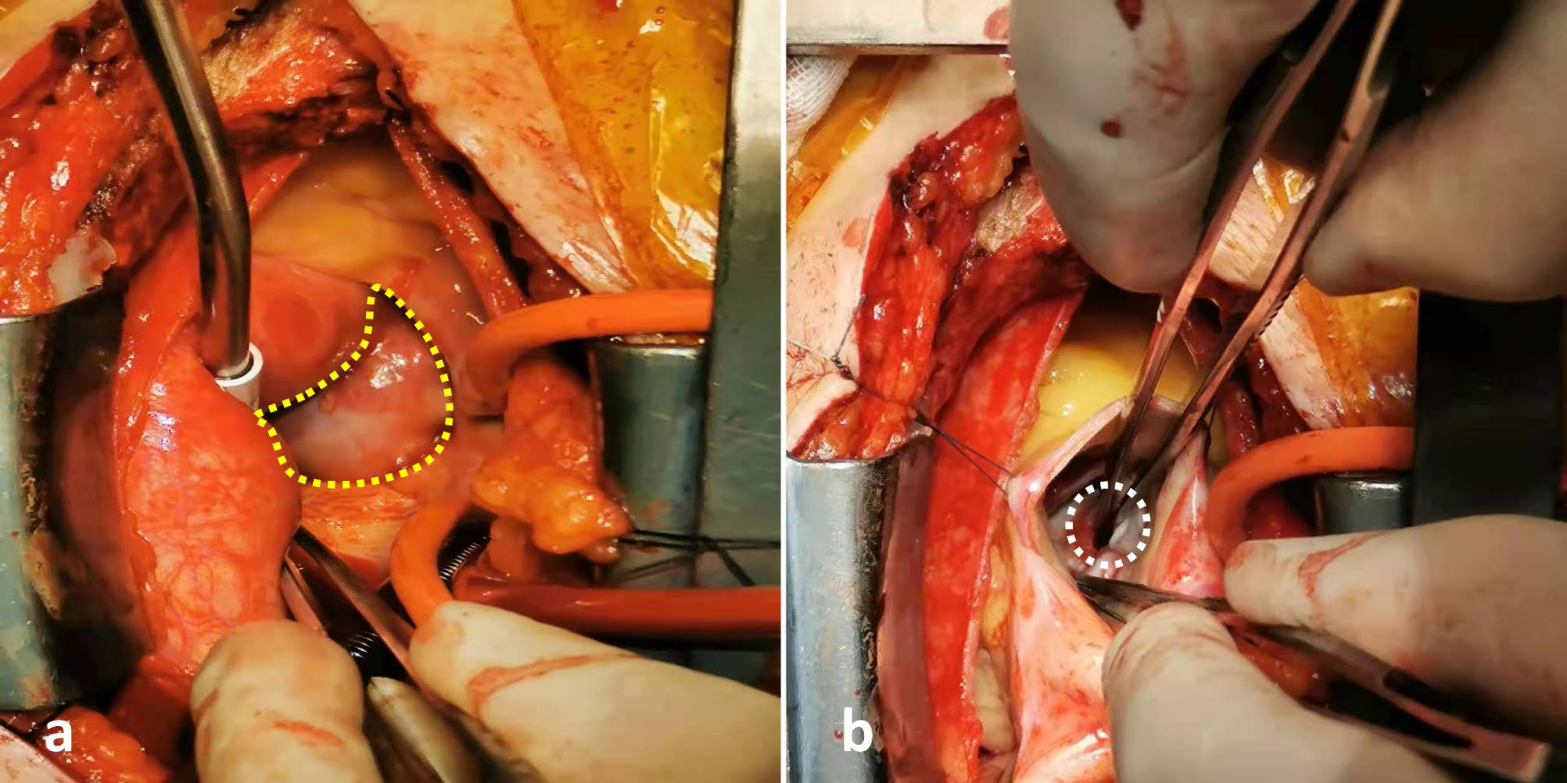
Surgical visual field. **a** Yellow dotted line indicates P-MAIVF; **b** White dotted line indicates the orifice of P-MAIVF.

The patient returned to our hospital a year later for a routine reexamination, and no complaint of special discomfort was mentioned. TTE demonstrated a pseudoaneurysm between the aortic root and the left atrium, with a range of 34 × 19 mm (Fig. 2), and contrast-enhanced CT further verified a pseudoaneurysm at the root of the ascending aorta (Fig. 3). Due to the risk of rupture of the pseudoaneurysm, the patient underwent surgery. During operation, the patient was monitored by TEE. The three-dimensional transesophageal echocardiography (3D-TEE) discovered a small orifice of pseudoaneurysm with a diameter of 3 mm. The orifice was located between the left coronary valve, non-coronary valve and anterior mitral valve (Fig. 4, Video. 3). The range of pseudoaneurysm measured by TEE was approximately 40 × 30 mm. Color Doppler flow imaging (CDFI) showed the blood flow into the pseudoaneurysm during systole and the blood flow back into the left ventricular outflow tract (LVOT) during diastole (Fig. 5, Video. 4). During the operation, a pseudoaneurysm was observed beneath the left atrium on the right side of the aortic root, with a range of 40 × 30 mm (Fig. 6). An irregular orifice with a maximum diameter of approximately 5 mm was observed below the junction between the left coronary artery valve and the non-coronary artery valve. After repairing the orifice of pseudoaneurysm, intraoperative TEE revealed that the orifice was closed and the arterial blood flow signals disappeared at the origin.

## Discussion and conclusions

P-MAIVF generally occurs in patients suffering from infectious endocarditis or after cardiac surgery. A pulsing echo-free sac that expands during systole and collapses during diastole is the remarkable feature of a pseudoaneurysm in echocardiography [2]. It usually remains asymptomatic unless complications arise. Severe dilation of P-MAIVF can compress the coronary artery. It could also rupture into the left atrium, forming a fistula between the LVOT and the left atrium, which can cause acute heart failure. Pericardial tamponade can occur if the pseudoaneurysm ruptures into the pericardial space, resulting in death.

In this case, the patient was initially diagnosed with myocardial infarction, and echocardiography showed no abnormalities in MAIVF. However, a subsequent TTE examination one year after PTCA revealed the presence of P-MAIVF. The patient had no surgical history and no evidence of infective endocarditis. After consulting with the surgeon, it was considered that the pseudoaneurysm may have been caused by the injury of the cardiac catheter while crossing the aortic annulus during the operation. Because of the weak structure there, a pseudoaneurysm gradually formed.

As P-MAIVF rupture into the pericardium can be fatal, surgery is recommended for all patients, even without symptoms. However, several reports have documented the effective percutaneous closure of P-MAIVF [3].

### Electronic supplementary material

Below is the link to the electronic supplementary material.

Supplementary Material 1

Supplementary Material 2

Supplementary Material 3

Supplementary Material 4

Supplementary Material 5

Supplementary Material 6

## Data Availability

The raw data could be contacted for Debo Xie who is the first author of this manuscript.

## List of abbreviations

MAIVF: Mitral-aortic intervalvular fibrosa
P-MAIVF: Pseudoaneurysm of the mitral-aortic intervalvular fibrosa
PTCA: Percutaneous transluminal coronary angioplasty
TTE: Transthoracic echocardiography
3D-TEE: Three-dimensional transesophageal echocardiography
CDFI: Color Doppler flow imaging
LVOT: Left ventricular outflow tract
