# Supplementary material for: Pseudoaneurysm of the mitral-aortic intervalvular fibrosa: a rare case after percutaneous transluminal coronary angioplasty
Source: BMC Cardiovasc Disord. 2023 Sep 26;23:477. doi: 10.1186/s12872-023-03512-4 (PMC10521427; doi:10.1186/s12872-023-03512-4)
Supplement: Supplementary file 4 — Supplementary Material 4 [file 12872_2023_3512_MOESM4_ESM.docx]

Video. 1 The coronary angio films during plasty.

Video. 2 The coronary angio films after plasty.

Video. 3 The P-MAIVF(*) is located near mitral valve and aortic valve.

Video. 4 2D shows P-MAIVF expands during systole and contracts during diastole.

Video. 5 CDFI shows the blood flow into the P-MAIVF during systole and the blood flow back into LVOT during diastole.
